# Longitudinal Pattern of Aerenchyma Formation Using the Ti-Gompertz Model in Rice Adventitious Roots

**DOI:** 10.3389/fpls.2021.776971

**Published:** 2021-11-30

**Authors:** Yun Chen, Guoming Li, Buhong Zhao, Yajun Zhang, Kun Liu, Priyadarshani Nadeeshika Samarawickrama, Xiaoxia Wu, Bing Lv, Lijun Liu

**Affiliations:** ^1^Jiangsu Key Laboratory of Crop Genetics and Physiology, Jiangsu Key Laboratory of Crop Genomics and Molecular Breeding, Jiangsu Co-Innovation Center for Modern Production Technology of Grain Crops, Yangzhou University, Yangzhou, China; ^2^College of Bioscience and Biotechnology, Yangzhou University, Yangzhou, China; ^3^Lixiahe Agricultural Institute of Jiangsu Province, Yangzhou, China

**Keywords:** rice root, aerenchyma, longitudinal pattern, Gompertz model, reparameterization

## Abstract

The longitudinal pattern of root aerenchyma formation of its relationship with the function of adventitious roots in rice remains unclear. In this study, the percentage of the aerenchyma area to the cross-sectional area (i.e., aerenchyma percentage) was fit with four non-linear models, namely, W_0_-Gompertz, Ti-Gompertz, logistic, and von Bertalanffy. Goodness-of-fit criteria such as the *R*^2^, the Akaike information criterion (AIC), and the Bayesian information criterion (BIC) were used to select the model. The bias of the parameters was evaluated using the difference between the ordinary least squares-based parameter estimates and the mean of 1,000 bootstrap-based parameter estimates and the symmetry of the distributions of these parameters. The results showed that the Ti-Gompertz model, which had a high goodness-of-fit with an *R*^2^ close to 1, lower AIC and BIC values, parameter estimates close to being unbiased, and good linear approximation, provided the best fit for the longitude pattern of rice aerenchyma formation with different root lengths among the competing models. Using the second- and third-order derivatives according to the distance from the root apex, the critical points of Ti-Gompertz were calculated. The rapid stage for aerenchyma formation was from the maximum acceleration point (1.38–1.76 cm from the root apex) to the maximum deceleration point (3.13–4.19 cm from the root apex). In this stage, the aerenchyma percentage increased by 5.3–15.7% per cm, suggesting that the cortical cells tended to die rapidly for the aerenchyma formation rather than for the respiration cost during this stage. Meanwhile, the volume of the aerenchyma of the entire roots could be computed using the integral function of the Ti-Gompertz model. We proposed that the longitudinal pattern of root aerenchyma formation modeled by the Ti-Gompertz model helped to deeply understand the relationship between the anatomical traits and physiological function in rice adventitious roots.

## Introduction

The cortical aerenchyma of adventitious roots is a fundamental anatomical trait across scales from root architecture to programmed cell death and physiological function (Drew et al., 2000; Lynch, 2011; Vanhees et al., 2020). Roots with greater gas-filled volume (i.e., greater aerenchyma or porosity) can achieve longer lengths in waterlogged soil than roots with lower aerenchyma and porosity (Armstrong, 1979; Armstrong and Beckett, 1987). Aerenchyma formation begins at the apical part of the roots and gradually grows toward the basal part of the rice adventitious roots, forming a longitudinal path for gas diffusion in flooded paddies (Armstrong, 1979; Colmer, 2003; Yamauchi et al., 2021). As the root apex grows farther away from the shoot base, root growth into anoxic waterlogged soils depends on the distance to which adequate oxygen can reach to sustain the respiratory demands of the root apex (Armstrong, 1979; Armstrong and Beckett, 1987). Thus, the aerenchyma formation might vary at different positions of the roots and with different root lengths and acts as a crucial index to evaluate the ability of oxygen transportation in rice roots and aerobic conditions in the rhizosphere (Yamauchi et al., 2019b; Chen et al., 2021).

At present, a lot of studies have reported the longitudinal pattern of the aerenchyma formation in the adventitious roots of rice (Yamauchi et al., 2016, 2019a). Rice has a fibrous root system consisting of a dense mass of adventitious roots (Meng et al., 2019). It is difficult to determine the aerenchyma formation along the entire root as preparing the cross-sections of roots and extracting regional data from root cross-sectional images are labor-intensive and have a low throughput (Takahashi and Pradal, 2021).

Recent improvements in plant modeling have allowed a deeper understanding of and more accurate predictions for a wide range of issues, such as competition among plants, plant-herbivore interactions, ecosystem functioning, and aerenchyma formation (Paine et al., 2012; Yamauchi et al., 2020). Previous studies analyzed the data on the aerenchyma area and distance from the root apex using bionomic smoothing (Karahara et al., 2012; Yukiyoshi and Karahara, 2014). They calculated the volume of aerenchyma in rice roots but did not reveal the developmental pattern of aerenchyma (Yukiyoshi and Karahara, 2014). The Gompertz model is well known and widely used in many aspects of biology (Gompertz, 1825; Archontoulis and Miguez, 2015; Tjørve and Tjørve, 2017). Yamauchi converted the distance from the root apex to time according to the root elongation rate and illustrated the age-dependent aerenchyma formation in rice roots by the Gompertz (i.e., W_0_-form of Gompertz) (Yamauchi et al., 2020, 2021).

However, the root elongation rate varies with the root diameter and the depth that the root apex penetrates into the impeded soil (Lynch et al., 2021; Takahashi and Pradal, 2021). More active growth occurs at the root apex over the mature root zone (Lynch et al., 2021). Previous studies have not considered on the effect of the root length or are restricted to a few lengths when modeling the longitudinal pattern of aerenchyma formation. Aerenchyma is fully developed in the mature root zone, whereas the root apex, where cell division and cell elongation occur, does not possess aerenchyma (Yamauchi et al., 2017, 2019a). Hence, the root elongation rate and the development of aerenchyma vary among roots with different root lengths, even within the same root type, which raises the question of how the longitudinal pattern of aerenchyma formation is uniformized with the root length.

The non-linear regression models, such as exponential models, growth models, and photosynthesis models, are widely applied in agricultural research (Archontoulis and Miguez, 2015). Fitting non-linear models is not a single-step procedure but an involved process that requires careful examination of each individual step (Paine et al., 2012). Finding the “best” model among competing models includes obtaining acceptable parameter estimates and a good model fit while meeting the standard assumptions of statistical models (Paine et al., 2012; Sari et al., 2019). Gompertz and logistic models, which contribute or facilitate the interpretation of the processes involved in plant growth, stand out; and sincere parameters allow efficient practical interpretations (Bem et al., 2017). However, there are two main types of Gompertz model forms, namely, W_0_-Gompertz and Ti-Gompertz model, with different location parameters; and the parameters are not easily interpreted if they are not converted to more useful measurements (Tjørve and Tjørve, 2017). Only W_0_-Gompertz has been used to fit the aerenchyma formation, and no further analysis about regression of the model has been conducted (Yamauchi et al., 2020). Furthermore, using a wider root length range yields a larger sample size and can increase the precision of the parameter estimates (Bohnstedt et al., 2021). It is also important to determine the biological meaning of the critical points in regression models in agriculture, as it allows one to adjust nutritional needs at the time when the growth rate is at its maximum, to determine the age at which an organism reaches maturity for harvesting, and to forecast crops in different scenarios (Pinho et al., 2014; Bem et al., 2017). Hence, selecting a suitable model and reparameterizing and determining the critical points in the aerenchyma formation modeling can help to elucidate the relationship between the development of aerenchyma and root physiological function.

In previous studies, less attention has been given to the development of aerenchyma over various root lengths and revealing the aerenchyma formation of the development process in adventitious roots. In this study, we used the rice cultivar Yangdao 6 as the material, prepared the cross-sections of adventitious roots with different lengths, and modeled the data of the percentage on the aerenchyma area to the cross-sectional area. The major objectives were to (1) screen the best model for describing the longitudinal pattern of the development of root aerenchyma, (2) elucidate the biological meaning of the proposed mathematical model of rice roots, and (3) develop a new method for the volume determination of the aerenchyma of rice roots. Our focus was mainly on the development process of adventitious roots with various root lengths and to achieve a real biologically improved model that predicts the aerenchyma formation in rice roots.

## Materials and Methods

### Plant Materials and Growth Conditions

*Indica* rice (*Oryza Sativa* L.) cultivar “Yangdao 6” was used as the material. The rice seeds were germinated and then grew in a growth chamber under 16-h light, 28°C, and 70% humidity using hydroponics. The composition of the nutrient solution is determined following the method proposed by Yoshida et al. (1976). The pH value was adjusted to 5.8, and the dissolved oxygen concentration was maintained at 6.5–7.0 mg L^–1^. Rice roots of 2-cm length from 20-day-old seedlings were marked using an oil-based marker pen. The marked roots were sampled for the anatomical observation after they grew to different lengths from 3 to 16 cm in the growth chamber.

### Analysis of the Anatomy of the Aerenchyma

Roots with different lengths were fixed in glutaraldehyde for 48 h and dehydrated in ethanol at the concentrations of 30, 40, 50, 60, and 70% for 20 min (each concentration). The cross-sections were prepared at every 0.5-cm interval from the root apex by hand sectioning with a razor blade under a pose microscope (Leica EZ24W, Germany) (Yamauchi et al., 2016). Each section was photographed using a microscope equipped with an AxioCam HRc camera (ZEISS, United States). All the images were analyzed by dividing the airspace area, and the total area of root sections and their areas were quantified with ImageJ (version 1.43) (Chen et al., 2021).

### Non-linear Regression Analyses

Four non-linear models were evaluated to describe the longitudinal pattern of aerenchyma formation. The mean values of the percentage of the aerenchyma area to the cross-sectional area (i.e., aerenchyma percentage) were fitted using *cftool* in MATLAB 2018a (MathWorks, Natick, MA, United States) with the following models:

(1) W-0Gompertz:y=ACe(-Bx)

(2) $$\mathrm{Ti}-\mathrm{Gompertz}:y={\mathrm{Ae}}^{\left(-{\text{e}}^{\left(-B\,\left(x-C\right)\right)}\right)}$$

(3) $$\mathrm{Logistic}:y=\frac{A}{1+e^{\left(C-\mathrm{Bx}\right)}}$$

(4) $$\mathrm{von}\,\mathrm{Bertalanffy}:y=A\,{\left(1-C\,{\text{e}}^{\left(\mathrm{Bx}\right)}\right)}^{3}$$

where *y* is the dependent variable (i.e., aerenchyma percentage) at the distances from the root apex, *x* is the distance from the root apex, *A* is the parameter that represents the asymptotic value, *B* is the parameter related to the growth rate, and *C* is the parameter that represents the distance between the initial value and the asymptote (Tjørve and Tjørve, 2017; Sari et al., 2019; Teixeira et al., 2021).

With *cftool* in the MATLAB software, *R*^2^ and the sum of squares of residuals (SSR) were obtained. The Akaike information criterion (AIC) and the Bayesian information criterion (BIC) were calculated as follows:

(5) $$\text{AIC}=n+\ln\left(\frac{\text{SSR}}{\text{n}}\right)+2\times p$$

(6) $$\mathrm{BIC}=n\,\times \ln\left(\frac{\text{SSR}}{n}\right)+p\,\times \ln\left(n\,\right)$$

where *R*^2^ is the coefficient of determination of the model, SSR is the sum of squares of residuals of the model, *p* is the number of the model parameters, AIC is the Akaike information criterion, and BIC is the Bayesian information criterion (Pinho et al., 2014).

The parameters of different models were estimated by the ordinary least squares (OLS) method and the bootstrap-based parameter estimation (Bates and Watts, 2007). In this procedure, the distribution plots of the obtained 1,000 bootstrap-based parameter estimates were constructed for each model, and the 95% CIs were computed as the difference between the 97.5th and 2.5th percentiles of 1,000 parameter estimates. The probability density function (short for density) is defined by the following formula: $P\left(a\leq x\leq b\right)=\int_{a}^{b}f\left(x\right)\text{dx}$, where (*a*, *b*) is the interval in which *x* lies, *P* (*a* ≤ *x* ≤ *b*) is the probability that some value of *x* lies within this interval, dx is *b*−*a*, and *f*(*x*) is density.

The bias of the parameters was investigated by using the following: (1) the degree of symmetry of the sampling distribution of parameter estimates and (2) the difference between the average mean of the bootstrap parameter estimates and the OLS estimates. Asymmetric distributions and high differences between the mean bootstrap estimates and the OLS estimates were related to biased parameters (Ritz and Streibig, 2008; Sari et al., 2019).

### Obtaining the Critical Points of the Selected Model

The abscissa values (i.e., distance from the root apex) of the critical points in the Ti-Gompertz model were calculated by the following equations (Bem et al., 2017; Tjørve and Tjørve, 2017).

The initial points (InP) were obtained by equaling the dependent variance (i.e., aerenchyma percentage) to 1 as follows:

(7) $$1=A\,e^{\left(-{\text{e}}^{\left(B\,\left(x-C\right)\right)}\right)}$$

The maximum value points (MVP) were obtained by setting the dependent variance (i.e., aerenchyma percentage) to the 95% estimated parameter *A* as follows:

(8) $$95\%\,A\,=A\,e^{\left(-{\text{e}}^{\left(B\,\left(x-C\right)\right)}\right)}$$

The inflection points (IPs) were set equal to the value of the parameter *C*.

The maximum acceleration points (MAPs) and the maximum deceleration points (MDPs) were obtained by equating the third-order derivative to zero as follows:

(9) $$\mathrm{The}\,\mathrm{abscissa}\,\mathrm{values}\,\mathrm{of}\,\mathrm{MAP}=\left(\frac{B\,C-\ln\left(2.618\right)}{B}\right)$$

(10) $$\mathrm{The}\,\mathrm{abscissa}\,\mathrm{values}\,\mathrm{of}\,\mathrm{MDP}=\left(\frac{B\,C-\ln\left(0.382\right)}{B}\right)$$

### Statistical Analysis and Software

The experimental data were sorted using Microsoft Excel 2013. The models were fit and plotted using *cftool* in MATLAB software. The non-linear regression analysis such as the OLS and the bootstrap-based parameter estimations was conducted using the *nlstools* package in R 4.0.2, and the graphs were constructed using the *ggplot2* package in R (Baty et al., 2015).

## Results

### Aerenchyma Formation in Rice Roots With Different Lengths

**FIGURE 1:**
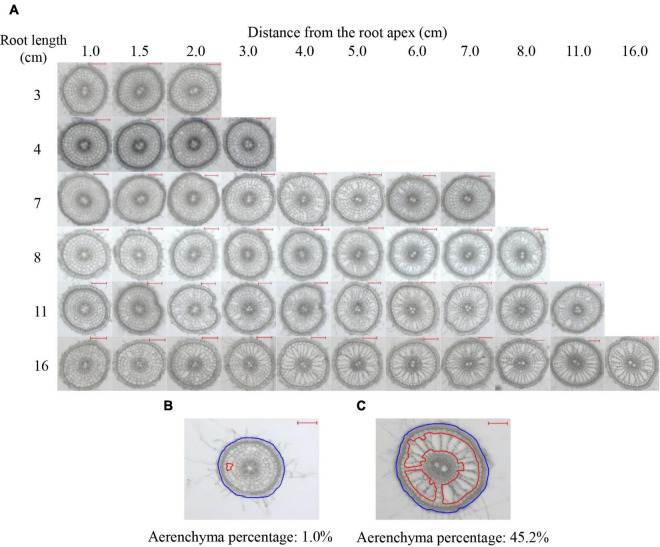
Typical cross-sections of rice adventitious roots with different lengths. **(A)** Pictures of the cross-sections of adventitious roots with lengths of 3, 4, 7, 8, 11, and 16 cm. **(B)** The minimum estimation of aerenchyma. **(C)** The maximum estimation of aerenchyma. The lacunae encircled by red lines denote the aerenchyma area of the root. The region encircled by blue lines denotes the cross-sectional area of the root. All lines were drawn using ImageJ software. Aerenchyma percentage: the percentage of aerenchyma area to the cross-sectional area; Bars = 200 μm.

Root cross-sections were prepared every 0.5 cm from the apex of 2- to 16-cm-long adventitious roots (Figure 1A). According to all values of the percentages of the aerenchyma area to the cross-sectional area (short for aerenchyma percentage), the minimum and maximum values were 1.0 and 45.2%, respectively, as observed in Figures 1B,C. No aerenchyma was observed in roots ≤3 cm. The aerenchyma began to form in roots ≥4 cm. The InP of aerenchyma formation were 1.5 and 1.0 cm from the root apex of 4- to 10-cm roots and 11- to 16-cm roots, respectively. The aerenchyma percentage gradually increased as the distance from the root apex in roots that were 4–7 cm in length increased. For roots ≥8 cm, the aerenchyma percentage increased and then reached a maximum level (Figures 1A–C and Supplementary Figure 1). Comparing the aerenchyma percentage at the same point from the apex of roots with different lengths, the longer the root length was, the more developed the aerenchyma (Figure 1A). Additionally, the aerenchyma percentage was lower at approximately 1 cm from the basal part of all roots (Figure 1A).

### Model Fit

**FIGURE 2:**
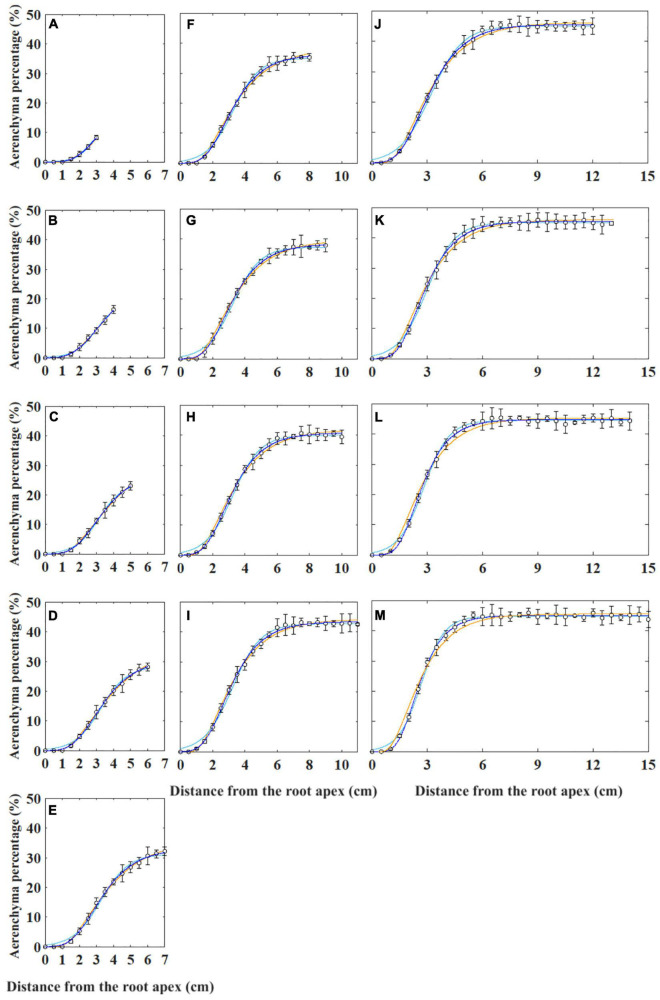
Non-linear models fitting for the percentage of aerenchyma area to the cross-sectional area (i.e., aerenchyma percentage) in rice adventitious roots with different lengths from 4 to 16 cm **(A–M)**. The hollow circles and bars represent the means of the observed aerenchyma percentage (*n* = 12). The lines in blue, sky blue, and orange represent the non-linear model fitting for the aerenchyma percentage with the Gompertz (i.e., W_0_-Gompertz and Ti-Gompertz), logistic, and von Bertalanffy models, respectively. The values of the 1-cm segment at the basal part of all roots were omitted.

Generally, the curves describing aerenchyma formation were S-shaped, except for the last 1 cm from the root apex (Supplementary Figure 1). The aerenchyma percentage data of the roots with different lengths were fit using four types of non-linear models such as two types of Gompertz (i.e., W_0_-Gompertz and Ti-Gompertz), logistic, and von Bertalanffy models (Figure 2). The W_0_-Gompertz model and the Ti-Gompertz model are the different reparameterization forms of the traditional Gompertz model. Our results showed that the fitted curves of the Ti-Gompertz and W_0_-Gompertz models were the same and coincided with the observed values (Figure 2 and Supplementary Figure 1). A logistic model fits the aerenchyma data of roots <7 cm well (Figures 2A–C), but the fitted curves showed differences from the observed values at 0–1 cm from the apex of roots ≥7 cm. The maximum fitted aerenchyma percentage was 2.0%, while the observed value was 0 (i.e., no aerenchyma formation was observed) (Figures 2D–M). The von Bertalanffy model fits the aerenchyma data of roots ≤13 cm well (Figures 2A–J) but showed the maximum difference between fitted and observed values by 2.6% at 1–6 cm from the apex of roots >13 cm (Figures 2K–M).

### Model Selection

**FIGURE 3:**
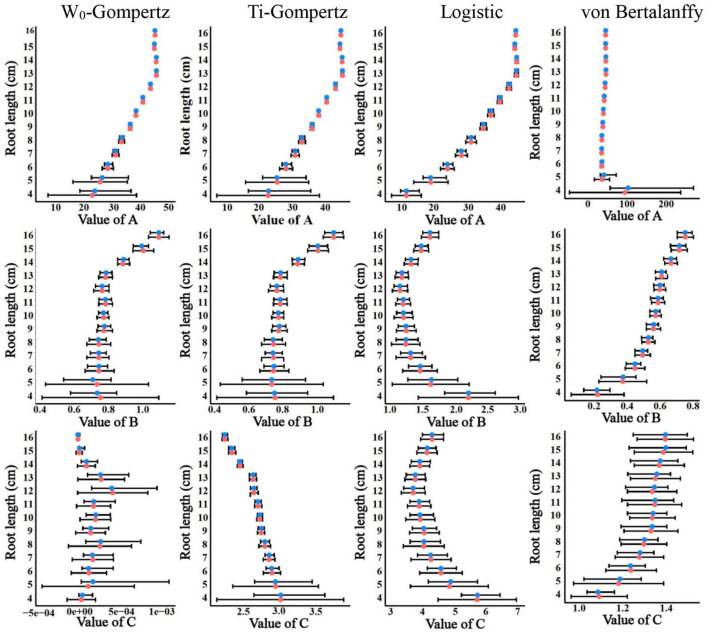
The estimates and their 95% CIs of the parameters *A*, *B*, and *C* obtained by the ordinary least squares (red points) and bootstrap-based estimates (blue points) of the W_0_-Gompertz, Ti-Gompertz, logistic, and von Bertalanffy models fit to the aerenchyma percentage in rice adventitious roots with different lengths.

**FIGURE 4:**
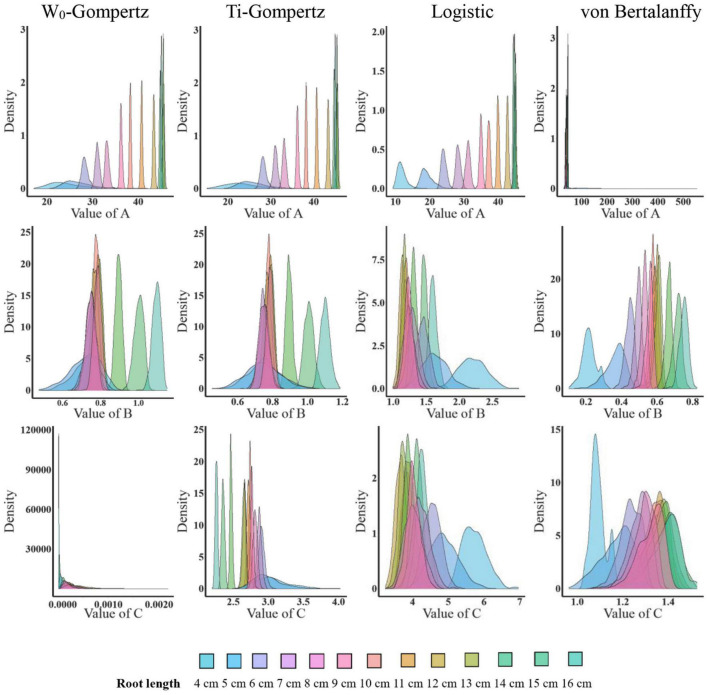
The distribution of the parameter estimates by the bootstrap-based parameter estimation of the W_0_-Gompertz, Ti-Gompertz, logistic, and von Bertalanffy models fit to the aerenchyma percentage in rice adventitious roots of different lengths. Density is the probability density function. The *nlsBoot()* function in the *nlstools* package in R was used to obtain 1,000 bootstrap estimates for each parameter.

Aiming to identify the best fit model, we used several procedures to check the goodness of fit and estimate the parameters. There was no difference between the W_0_-Gompertz and Ti-Gompertz models based on the observed criteria. The *R*^2^ values were greater than 0.99 (close to 1) for all models. Compared with logistic and von Bertalanffy models, two Gompertz models showed the lowest values for the Akaike information criterion (AIC) and the Bayesian information criterion (BIC) within the same root length. Overall, there was a good adjustment of the investigated models, and the Gompertz models produced the best fit of the data for the aerenchyma percentage based on the three criteria (Supplementary Table 1). Furthermore, the difference in the estimates between the OLS and bootstrap-based approaches in the four models showed less bias for roots ≥6 cm (Figure 3). The bias of the estimates and the 95% CIs of the parameters for roots <6 cm among the four models were different (Figure 3). The W_0_-Gompertz and von Bertalanffy models had larger biases and CIs than the other models (Figure 3). The Ti-Gompertz and logistic models had parameter estimates close to being unbiased, and all assumptions were met (Figure 4). Most of the distributions of the parameters were symmetrical except that parameter *A* in the von Bertalanffy model and parameter *C* in the W_0_-Gompertz model were asymmetrical (Figure 4).

Consequently, the Ti-Gompertz model had higher goodness of fit, lower difference between parameter estimates by the OLS and bootstrapping, and symmetrical distribution of all parameters. This was a direct result of the good linear approximation of the Ti-Gompertz model compared to other models, suggesting that the Ti-Gompertz model was the best to describe the data of the aerenchyma formation.

### The Unified Parameters in the Ti-Gompertz Model

**FIGURE 5:**
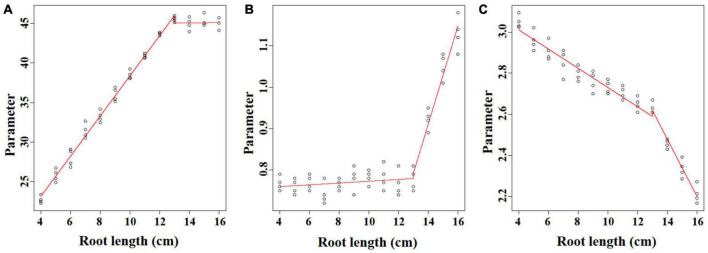
The linear regression analysis of the parameters **(A–C)** of the Ti-Gompertz model with the root length. The model was characterized by two phases with root lengths ≤13 cm and ≥13 cm. The values of the parameters were obtained by fitting the aerenchyma percentage of each root length with the Ti-Gompertz model. The experiment was replicated four times (*n* = 4). The *preview()* function in the *nlstools* package in R was used to plot the graph.

The linear regression analysis was performed to unify the Ti-Gompertz model for roots with different lengths. Parameter *A* represents the asymptotic maximum aerenchyma percentage. For roots <13 cm, the values of the asymptotes increased as the root length (*L*) increased, and the equation was *A* = 13.02 + 2.54 *L* (*P* < 0.001; Figure 5A). The maximum aerenchyma percentage was 44.8 for all roots ≥13 cm and showed no correlation with the root length (*A* = 44.8 − 0.021 *L*; *P* = 0.934; Figure 5A). Parameter *B* represents the maximum speed of aerenchyma formation. For roots <13 cm, *B* had no correlation with the root length, and the maximum speed of aerenchyma formation was 0.751 (*B* = 0.751 + 0.0022 *L*; *P* = 0.222; Figure 5B). For roots ≥13 cm, *B* had a significantly positive correlation with the root length (*B* = −0.748 + 0.119 *L*; *P* < 0.001; Figure 5B). Parameter *C* represents the distance from the root apex when the aerenchyma formation speed reaches a maximum. *C* showed a significantly negative correlation with the root length, and the equations were *C* = 3.2 − 0.047 *L* (*L* < 13; *P* < 0.001) and *C* = 4.43 − 0.139 *L* (*L* ≥ 13; *P* < 0.001) (Figure 5C).

### Describing Aerenchyma Formation Using the Ti-Gompertz Model

According to the minimum value of 1.0% for the aerenchyma percentage, we calculated the abscissa value of the initial point of aerenchyma formation under a dependent variance (i.e., aerenchyma percentage) of 1.0%. The coordinates of MAPs, IPs, and MDPs of the Ti-Gompertz model were obtained using the second- and third-order partial derivatives. The abscissa values of the MAPs, IPs, and MDPs appeared at 1.38–1.76 cm, 2.26–2.96 cm, and 3.13–4.19 cm from the root apex, respectively, and decreased as the root length increased (Table 1). The development of aerenchyma increased exponentially and logarithmically from the MAP to the IP and from the IP to the MDP, respectively (Table 1). These critical points in the Ti-Gompertz model suggested that the longer the roots were, the more developed the aerenchyma at the same point from the apex of roots with different lengths.

**TABLE 1:** The abscissa values (distance from the root apex) of the critical points for the Ti-Gompertz model.

Root length	InP	MAP	IP	MDP	95% MVP
4 cm	1.53	1.76	3.03	–	–
5 cm	1.37	1.66	2.96	–	–
6 cm	1.30	1.63	2.91	4.19	–
7 cm	1.22	1.58	2.87	4.16	–
8 cm	1.14	1.52	2.81	4.10	6.78
9 cm	1.13	1.54	2.76	3.99	6.55
10 cm	1.08	1.50	2.74	3.98	6.56
11 cm	1.06	1.50	2.72	3.94	6.48
12 cm	0.94	1.41	2.66	3.91	6.52
13 cm	0.96	1.43	2.65	3.86	6.4
14 cm	0.97	1.39	2.47	3.55	5.79
15 cm	1.03	1.40	2.36	3.31	5.30
16 cm	1.04	1.38	2.26	3.13	4.96

*InP, initial point; MAP, maximum acceleration point; IP, inflection point; MDP, maximum deceleration point; 95% MVP, 95% of the maximum value point.*

**FIGURE 6:**
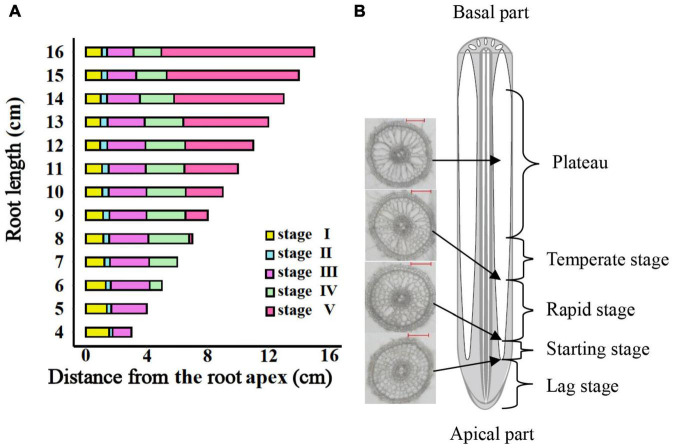
The entire period of aerenchyma formation in rice roots. **(A)** Five stages of aerenchyma formation divided according to the critical points of the Ti-Gompertz model. **(B)** The longitudinal pattern of a typical rice root. Stage I: the lag stage; Stage II: the starting stage (from the initial point to maximum acceleration); Stage III: the rapid stage (from maximum acceleration point to maximum deceleration point); Stage IV: the temperate stage (from maximum deceleration point to 95% maximum value point); and Stage V: the plateau (upper asymptote).

**FIGURE 7:**
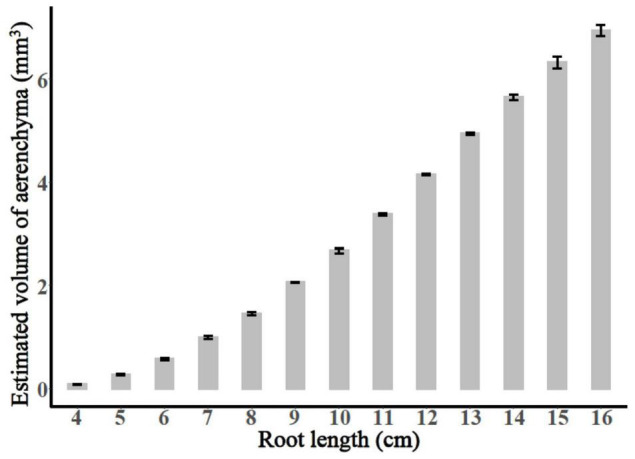
The calculated total volume of aerenchyma of the adventitious roots with different lengths.

The entire period of aerenchyma formation was further divided into five stages as follows: the lag stage (Stage I), the starting stage (Stage II), the rapid stage (Stage III), the temperate stage (Stage IV), and the plateau (Stage V) (Figure 6). Overall, for adventitious roots <11 cm, there was no aerenchyma formation at <1.0 cm from the root apex (Figure 1). Stage II was defined as from the InP to the MAP, at which the aerenchyma percentage increased by 2.8–6.7% per cm. Stage III started at 1.38–1.76 cm from the root apex and continued to grow for approximately 2.0 cm, at which the aerenchyma percentage increased by 5.3–15.7% per cm. Stage IV was defined as from the MDP to the 95% MVP, at which the aerenchyma increased 3.3–6.8% per cm. For adventitious roots ≥8 cm, the aerenchyma formation reached a plateau at which point the aerenchyma percentage reached its maximum (Figure 2, Table 1, and Supplementary Figure 2). The initial point of the plateau was close to the root apex as the root length increased (Table 1). Furthermore, using the integral function of the Ti-Gompertz model, the volume of the aerenchyma was computed with the length and average diameter of the roots implying that once the root length and diameter were determined, the volume of the aerenchyma in the entire roots could be further computed (Figure 7).

## Discussion

### The Ti-Gompertz Model Provides the Best Fit for the Longitudinal Pattern of Aerenchyma Formation in Rice Roots

Models are essential in biological science to summarize the data, to allow rapid predictions, and to design processes, such as “what-if” scenarios (Dolan and Mishra, 2013). Researchers are convinced that the age-dependent aerenchyma formation may not be the same for different root elongation rates, even though the patterns of distance-dependent aerenchyma formation are exactly the same; and the W_0_-Gompertz model will be useful for describing longitudinal patterns (Yamauchi et al., 2021).

However, regardless of whether the elongation rates among different roots were the same, we could still not conclude that the aerenchyma formation had the same pattern at the same position from the root apex or at the same time points after root cell emergence within a root system. In this study, we observed that aerenchyma formation varied at the same distance from the root apex in rice roots with different lengths; and the longer the root was, the higher the aerenchyma percentage (Figure 1 and Supplementary Figure 1). The maximum aerenchyma percentage did not increase if the root length was >13 cm on the plateau (Supplementary Figure 1). Therefore, the longitudinal pattern of aerenchyma formation in rice adventitious roots must be closely related to the root length, and it is practicable to unify the convinced pattern by mathematically modeling in the development process of adventitious roots with different root lengths.

Mostly, the selection of non-linear models has been performed using the goodness-of-fit measures such as *R*^2^, AIC, and BIC (Lucio et al., 2015; Bem et al., 2017), but these measures do not assess the biases of the parameters and may lead to selecting models that do not describe the biological phenomena correctly (Bates and Watts, 2007; Sari et al., 2019). Actually, when fitting the longitudinal pattern of aerenchyma formation with four types of non-linear models, all models had a good fit with an *R*^2^ > 0.99 (Supplementary Table 1). However, the lower difference between the average mean of the bootstrap parameter estimates and the OLS estimates and the symmetrical distribution of these estimates were only observed in the Ti-Gompertz model rather than the W_0_-Gompertz and other tested models (Figures 3, 4). Thus, the Ti-Gompertz model was the best for describing the longitudinal pattern of aerenchyma formation with different root lengths. Finally, according to the linear correlation of the parameters with the root length, we proposed the normalized Ti-Gompertz model as the following equation:

(11) $$\text{AR}=\left\{ \begin{array}{c} \text{if}\,L<13:\left(13.02+2.54\,\,L\right)\,e^{\left(-{\text{e}}^{\left(-0.751\,\left(D-\left(3.20-0.047\,L\right)\right)\right)}\right)}\\ \text{if}\,L\geq 13:44.8\,A\,e^{\left(-{\text{e}}^{\left(-\left(-0.748+0.119\,\,L\right)\,\left(D-\left(4.43-0.139\,L\right)\right)\right)}\right)} \end{array}\right.$$

where AR is the percentage of the aerenchyma area to the cross-sectional area in the rice roots, *D* is the distance from the root apex, and *L* is the root length.

With the longitudinal patterns displayed by different root lengths, it was generally observed that longer roots displayed more developed aerenchyma than shorter roots in Eq. (11) and Supplementary Figure 2.

### Biological Exploration of Aerenchyma Formation Based on the Parameters and Critical Points

Parameter estimation can be defined as “a discipline that provides tools for the efficient use of the data in the estimation of constants appearing in mathematical models and for aiding in modeling of phenomena” (Dolan and Mishra, 2013). However, the two Gompertz forms were essentially the same model in mathematics (Tjørve and Tjørve, 2017). Therefore, we switched the equation form from the W_0_-Gompertz model to the Ti-Gompertz model and reparameterized the Ti-Gompertz model. Apparently, compared with the W_0_-Gompertz model, the Ti-Gompertz model provided parameter estimates close to being unbiased and good linear approximation; hence, this conversion made it possible to correlate root aerenchyma formation with any length of adventitious roots (Eq. (11); Figure 2).

Furthermore, determining the critical points and their biological meanings of models (e.g., Gompertz and logistic) is important in agricultural work (Ersoy et al., 2006; Prado et al., 2013; Pinho et al., 2014; Archontoulis and Miguez, 2015). The value at the IP of the sigmoid curve of the Gompertz model is locked at 36.8% of the upper asymptote (MVP) (Pinho et al., 2014; Tjørve and Tjørve, 2017). Around the IP, from the MAP to the MDP, the aerenchyma developed rapidly (Figures 2, 6). The asymptote increased and the IP decreased as the root length increased, also demonstrating that the development of aerenchyma in longer roots was stronger than that in shorter roots at the same position from the apex (Figure 2 and Supplementary Figure 1).

Several studies reported that the development of root aerenchyma could reduce root metabolic costs, and roots with large amounts of aerenchyma had lower respiration rates on a volume basis (Colmer, 2003; Zhu et al., 2010; Postma and Lynch, 2011; Saengwilai et al., 2014; Chimungu et al., 2015). Therefore, the aerenchyma formation improved plant performance under suboptimal nutrient and water availability and had a positive effect on overall root growth (Evans, 2004; Zhu et al., 2010; Jaramillo et al., 2013; Schneider et al., 2017). We described five stages of the Ti-Gompertz model based on the critical points and found that the speed of Stage III in the development of aerenchyma reached a maximum of 5.3–15.7% per cm and remained at 1.38–1.76 cm (Figure 6 and Table 1). It can be concluded that the cortical cells tended to die rapidly for the aerenchyma formation rather than for the respiration cost during this stage, and many anabolism processes were active. Thus, the cortical aerenchyma formation of rice adventitious roots at different longitudinal positions rather than that of the entire root might be a useful and precise trait for physiological functions such as gas transportation and water and nutrient acquisition. Eq. (11) of the Ti-Gompertz model, which we proposed in this study, helped to deeply understand the relationship between the trait and function of anatomy for rice adventitious roots.

To pinpoint the usefulness of the proposed model and the given results, we focused on some applications to the real data, and the volume of the entire aerenchyma could be calculated using the integral function of the Ti-Gompertz model (Figure 7). The method reported previously for the volume of the intercellular gas-filled spaces in roots measured the root porosity, which can be further enhanced by the aerenchyma formation (Jensen et al., 1969; Colmer, 2003). This study also reported alternative mathematical modeling for conveniently estimating the volume of the aerenchyma of the entire roots.

## Conclusion

The Ti-Gompertz model was found to be the most appropriate model in describing the aerenchyma formation curve in the development process of adventitious roots. Both the regression and reparameterization of the model showed that the Ti-Gompertz model had parameter estimates close to being unbiased and good linear approximation. We investigated the effect of the root length and the distance from the root apex on the aerenchyma formation through nested models [Eq. (11)]. Using the parameters and critical points of the model, it was possible to determine not only the longitudinal development process but also the relationship with the physiological function of rice root aerenchyma. In addition, we suggested that the Ti-Gompertz model may be an interesting alternative to porosity measurements to analyze the volume of rice roots. The procedures described in this study may contribute to the assessment of plant anatomical phenotype in precisely regulating the morphology and physiology of rice roots.

However, the root anatomical phenotypes are dynamic and respond to genotype and environmental changes (Vanhees et al., 2020). The modeling curves that were determined with a rice cultivar, i.e., Yangdao 6, under natural conditions herein might differ according to cultivar and rice growth conditions, and such variation might reduce the precision with which the longitudinal developmental curves can be assessed. Once the parameters are found to also vary with environmental conditions, the equation of the achieved model can help identify how the physiological characteristics are modulated by the aerenchyma formation responding to the environment. Thus, future studies might consider the longitudinal pattern for a larger set of genotypes and under varying conditions. Additional insight into the parameter estimation gained from *in silico* analysis and how parameters change during the aerenchyma formation is valuable. Furthermore, it is necessary to converge the effect of changing root aerenchyma characteristics to the physiological properties of roots.

## Data Availability Statement

The original contributions presented in the study are included in the article/Supplementary Material, further inquiries can be directed to the corresponding author.

## Author Contributions

YC, GL, and LL came up with the ideas and conceived the study. YC, GL, BZ, and YZ designed the experiments. YC, GL, YZ, KL, PN, XW, and BL performed the experiments and analyzed the data. YC, GL, and LL wrote the manuscript. XW helped to discuss the manuscript. All authors contributed to the manuscript.

## Conflict of Interest

The authors declare that the research was conducted in the absence of any commercial or financial relationships that could be construed as a potential conflict of interest.

## Publisher’s Note

All claims expressed in this article are solely those of the authors and do not necessarily represent those of their affiliated organizations, or those of the publisher, the editors and the reviewers. Any product that may be evaluated in this article, or claim that may be made by its manufacturer, is not guaranteed or endorsed by the publisher.
