# Supplementary figures and images for: Longitudinal Pattern of Aerenchyma Formation Using the Ti-Gompertz Model in Rice Adventitious Roots
Source: Front Plant Sci. 2021 Nov 30;12:776971. doi: 10.3389/fpls.2021.776971 (PMC8669396; doi:10.3389/fpls.2021.776971)

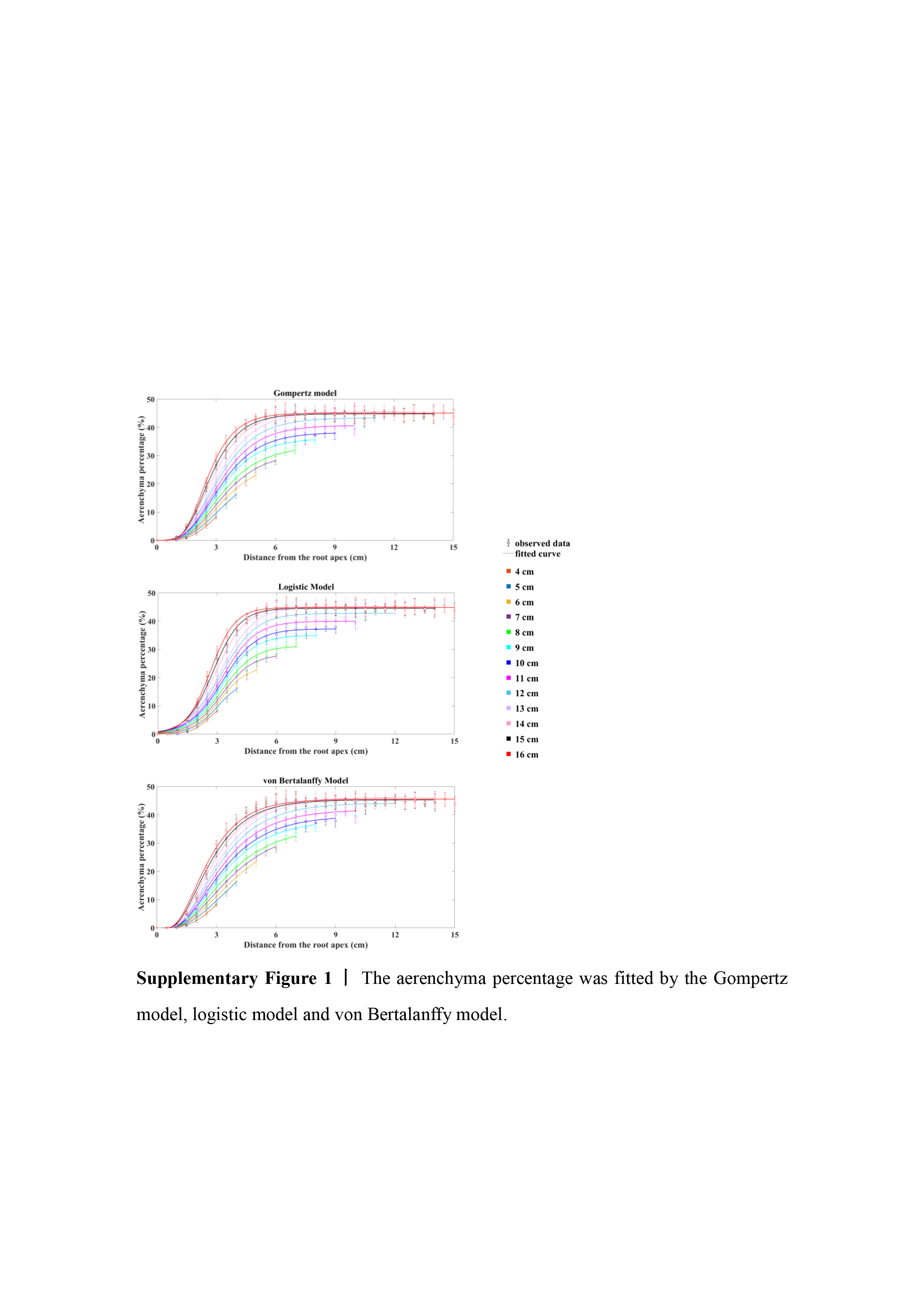

Supplement: Supplementary file 1 [file Image_1.jpg]

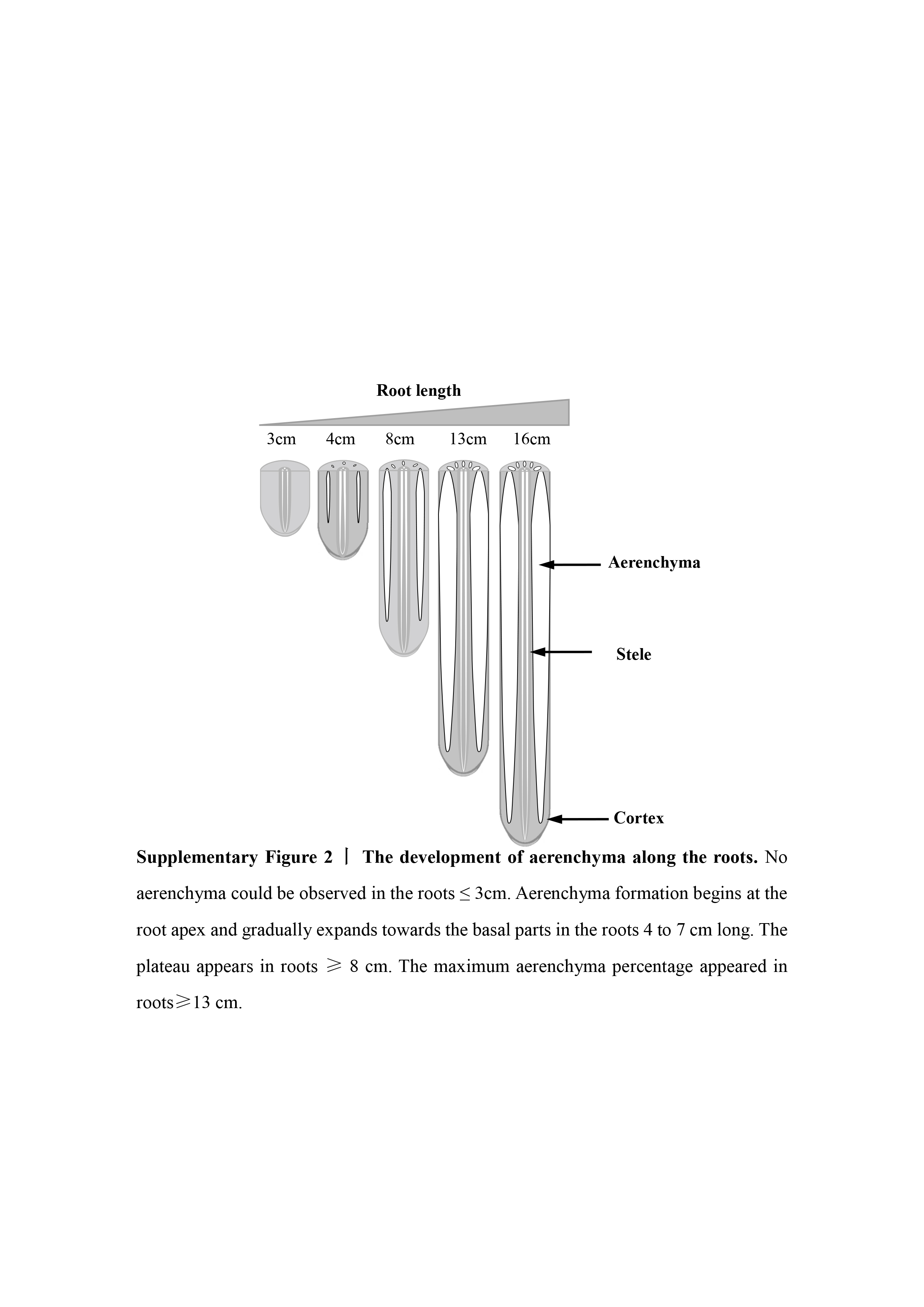

Supplement: Supplementary file 2 [file Image_2.jpg]
