# Supplementary material for: Longitudinal Pattern of Aerenchyma Formation Using the Ti-Gompertz Model in Rice Adventitious Roots
Source: Front Plant Sci. 2021 Nov 30;12:776971. doi: 10.3389/fpls.2021.776971 (PMC8669396; doi:10.3389/fpls.2021.776971)
Supplement: Supplementary file 3 [file Table_1.DOCX]

**Supplementary TABLE 1丨** Summary of the criteria values for comparing the Gompertz, logistic and von Bertalanffy models fitting for the aerenchyma percentage in rice adventitious roots with different lengths.

| Root length | R^2^ | | | AIC | | | | BIC | | | | |
| --- | --- | --- | --- | --- | --- | --- | --- | --- | --- | --- | --- | --- |
|  | G (W_0_/Ti) | L | VB | | G (W_0_/Ti) | L | VB | | G (W_0_/Ti) | L | VB |  |
| 4 cm | 0.999 | 0.997 | 0.999 | | -26.43 | -19.21 | -29.41 | | -26.59 | -19.41 | -29.57 |  |
| 5 cm | 0.996 | 0.991 | 0.997 | | -12.53 | -4.75 | -15.09 | | -11.94 | -4.15 | -14.50 |  |
| 6 cm | 0.999 | 0.996 | 0.999 | | -26.41 | -7.68 | -20.11 | | -25.22 | -6.49 | -18.91 |  |
| 7 cm | 0.999 | 0.995 | 0.999 | | -24.84 | -0.85 | -16.62 | | -23.15 | 0.85 | -14.92 |  |
| 8 cm | 0.999 | 0.993 | 0.998 | | -18.70 | 7.03 | -15.19 | | -16.58 | 9.16 | -13.06 |  |
| 9 cm | 0.999 | 0.996 | 0.997 | | -25.52 | 2.22 | -3.33 | | -23.02 | 4.72 | -0.83 |  |
| 10 cm | 0.999 | 0.995 | 0.998 | | -32.80 | 6.50 | -6.57 | | -29.97 | 9.39 | -3.74 |  |
| 11 cm | 0.999 | 0.997 | 0.996 | | -24.00 | 2.29 | 4.85 | | -20.87 | 5.42 | 7.98 |  |
| 12 cm | 0.999 | 0.996 | 0.997 | | -18.10 | 9.75 | 2.44 | | -14.69 | 13.15 | 5.84 |  |
| 13 cm | 0..999 | 0.997 | 0.996 | | -20.86 | 5.75 | 8.47 | | -17.20 | 9.41 | 12.13 |  |
| 14 cm | 0.999 | 0.997 | 0.995 | | -32.73 | -0.64 | 12.63 | | -28.84 | 3.24 | 16.51 |  |
| 15 cm | 0.998 | 0.997 | 0.992 | | -17.58 | -5.84 | 25.98 | | -13.48 | -1.74 | 30.08 |  |
| 16 cm | 0.999 | 0.997 | 0.992 | | -28.95 | -3.48 | 27.42 | | -24.65 | 0.82 | 31.72 |  |

G (W_0_/Ti): Gompertz models including W_0_-Gompertz and Ti-Gompertz; L: logistic model; VB: von Bertalanffy model. R^2^: coefficient of determination; AIC: Akaike information criterion; BIC: Bayesian information criterion.
